# Levels of Acylcarnitines and Branched-Chain Amino Acids in Antipsychotic-Treated Patients with Paranoid Schizophrenia with Metabolic Syndrome

**DOI:** 10.3390/metabo12090850

**Published:** 2022-09-09

**Authors:** Irina A. Mednova, Alexander A. Chernonosov, Elena G. Kornetova, Arkadiy V. Semke, Nikolay A. Bokhan, Vladimir V. Koval, Svetlana A. Ivanova

**Affiliations:** 1 Mental Health Research Institute, Tomsk National Research Medical Center, Russian Academy of Sciences, Aleutskaya Str. 4, 634014 Tomsk, Russia; 2 Institute of Chemical Biology and Fundamental Medicine, Siberian Branch of Russian Academy of Sciences, Lavrentyev Avenue 8, 630090 Novosibirsk, Russia; 3 Siberian State Medical University Hospital, Moskovsky Trakt 2, 634050 Tomsk, Russia; 4 Department of Psychiatry, Addictology and Psychotherapy, Siberian State Medical University, Moskovsky Trakt 2, 634050 Tomsk, Russia

**Keywords:** acylcarnitine, amino acid, schizophrenia, metabolic syndrome, metabolomics

## Abstract

Several studies have shown that patients with schizophrenia are at high risk for metabolic syndrome (MetS) and bioenergetic dysfunction. Because acylcarnitines are involved in bioenergetic pathways and reflect the functioning of mitochondria, we hypothesized that these compounds are biomarkers of MetS in schizophrenia. The aim of this work was to quantify acylcarnitines and branched-chain amino acids in patients with schizophrenia comorbid with MetS. The study included 112 patients with paranoid schizophrenia treated with antipsychotics. Among them, 39 subjects met criteria of MetS. Concentrations of 30 acylcarnitines and three amino acids in dry serum spots were measured by liquid chromatography coupled with tandem mass spectrometry. MetS patients were found to have higher levels of valeryl carnitine (C5), leucine/isoleucine, and alanine as compared with patients without MetS, indicating possible participation of these compounds in the pathogenesis of metabolic disorders in schizophrenia. In patients with paranoid schizophrenia with or without MetS, lower levels of carnitines C10, C10:1, C12, and C18 were recorded as compared with the healthy individuals (n = 70), implying deterioration of energy metabolism. We believe that this finding can be explained by effects of antipsychotic medication on an enzyme called carnitine-palmitoyl transferase I.

## 1. Introduction

Currently, there is no comprehensive hypothesis that can explain the pathophysiological mechanisms underlying clinical manifestations of negative and positive symptoms and of cognitive dysfunction in schizophrenia. Both genetic [1] and nongenetic factors contribute to the development of schizophrenia, including impaired neurotransmission [2], mitochondrial dysfunction [3], and low-grade inflammation [4,5]. To alleviate the symptoms of schizophrenia, patients are prescribed antipsychotic drugs—whose targets are neurotransmitter systems—almost for life. On the other hand, the use of antipsychotics often has metabolic adverse effects. The prevalence of metabolic syndrome (MetS) in antipsychotic-treated patients is significantly higher than in antipsychotic-naïve patients and ranges within 32.0–68.0% or 3.3–26.0%, respectively [6]. These data suggest that these psychotropic drugs affect brain areas involved in the regulation of the energy balance and metabolism [7]. In addition to the central mechanisms, there are also peripheral ones behind the development of antipsychotic-induced metabolic disorders [8,9,10,11].

Carnitines are important regulators of lipid metabolism and are responsible for the transport of long-chain fatty acids into the mitochondrial matrix and thus promote β-oxidation and energy production in the Krebs cycle. Additional functions of the carnitine system were described relatively recently, including the removal of excess acyl groups from the body, modulation of intracellular homeostasis of coenzyme A (CoA) [10], antioxidant effects [12], participation in the regulation of immune functions [13,14], and changes in cholinergic neurotransmission [15]. Acylcarnitines may be a link between metabolic and psychiatric disorders; an imbalance within the pool of acylcarnitines has been found in people with insulin-resistant obesity, diabetes mellitus, and MetS [16,17]. In addition, acylcarnitines have become a more accurate prognostic marker of type 2 diabetes mellitus [18] and its cardiovascular complications [19] as compared to disease risk factors. A connection has been identified between branched-chain amino acids and acylcarnitines having an odd number of carbon atoms [20]. An increase in the concentration of branched-chain amino acids is considered a marker of insulin resistance [20,21,22]. Alanine can be synthesized from pyruvate and branched-chain amino acids and is associated with several MetS-related traits, including the body–mass index, waist circumference, triglycerides, hypertension, impaired glucose tolerance, and insulin resistance. Alanine levels are elevated in obese Japanese populations [22].

Mechanisms of accumulation of acylcarnitines in metabolic disorders are explained as follows. Under conditions of excessive lipid availability, there is a discrepancy between the activity of β-oxidation and that of the tricarboxylic acid cycle. In this case, mitochondria are overloaded with an excess of fatty acids, which leads to their incomplete β-oxidation and manifests itself in the accumulation of fatty acids and their derivatives (acylcarnitines, ceramides, and diacylglycerides) in tissues and blood. This problem hampers switching between available oxidation substrates and promotes the development of insulin resistance. Elevated fatty acid oxidation overwhelms the ability of the tricarboxylic acid cycle to degrade acetyl-CoA. This anomaly results in a reverse β-oxidation reaction and accumulation of short-chain acylcarnitines, which may also contribute to insulin resistance. Another possible hypothesis explaining the development of insulin resistance is based on the ability of acylcarnitines to inhibit insulin signaling pathways by activating inflammation, for example, by inducing the expression of cyclooxygenase 2 or stimulating the secretion of proinflammatory cytokines [23,24,25,26,27]. On the other hand, under conditions of insulin resistance, there is greater formation of malonyl-CoA and inhibition of carnitine palmitoyltransferase I; this aberration leads to a decrease in the formation of ATP and is accompanied by lipogenesis with the formation of mainly triglycerides [17,28].

Previously, we and other researchers have shown changes in the profile of acylcarnitines and amino acids in patients with chronic or first-episode schizophrenia [29,30,31,32,33]. Meanwhile, data from only a single report are available where the profile of acylcarnitines was assessed depending on MetS. Cao B. et al. (2020) showed higher levels of 17 plasma acylcarnitines in patients with schizophrenia comorbid with MetS in comparison with the patients without MetS [30]. Unfortunately, those researchers did not assess levels of branched-chain amino acids, which may play a role in the development of metabolic disorders, as described above.

Thus, the aim of our project was to quantify acylcarnitines and branched-chain amino acids in patients with paranoid schizophrenia comorbid with MetS.

## 2. Materials and Methods

### 2.1. The Study Population and Sample Collection

The study was conducted in accordance with the Declaration of Helsinki of the World Medical Association. The study protocol was approved by the Ethics Committee of the Mental Health Research Institute, Tomsk National Research Medical Center, the Russian Academy of Sciences (approval of 24 April 2018, # 187). Before the onset of the study, informed consent was obtained from all patients and healthy individuals. This work included 112 patients with paranoid schizophrenia according to the International Statistical Classification of Diseases and Related Health Problems, 10th Revision (ICD-10: F20) at ages 18–55 years. MetS was diagnosed in accordance with the International Diabetes Federation criteria. According to this classification, MetS is diagnosed when there is central obesity (waist circumference greater than 94 and 80 cm in males and females, respectively) and at least two of the following criteria are fulfilled: (i) triglyceride concentration in blood serum is higher than 1.7 mmol/L (150 mg/dL) or lipid-lowering therapy is administered, (ii) high-density lipoprotein cholesterol concentration in blood serum is below 1.03 mmol/L (40 mg/dL) in males and 1.29 mmol/L (50 mg/dL) in females; (iii) arterial blood pressure is above 130/85 mmHg (or previously diagnosed hypertension is being treated), and (iv) glucose concentration in blood serum is higher than 5.6 mmol/L (100 mg/dL; or previously diagnosed type 2 diabetes mellitus is present) [34].

Blood samples were drawn after a 12 h overnight fast in the first days of hospitalization before antipsychotic treatment and were centrifuged for 30 min at 2000× *g* and 4 °C to isolate serum; the latter was stored at −80 °C until analysis. The serum was spotted on ProteinSaver card 903™ (Whatman^®^, Maidstone, UK) followed by air-drying at room temperature for at least 3 h before analysis.

### 2.2. Laboratory Measurements

Laboratory criteria defining the development of MetS, including levels of glucose, triglycerides, and high-density lipoprotein cholesterol, were determined in serum by standard biochemical methods using Cormay kits (Lomianki, Poland).

Concentrations of amino acids (alanine, valine, and leucine/isoleucine) and acylcarnitines (C0–C18) in the serum spots were measured by liquid chromatography coupled with tandem mass spectrometry by means of the Amino Acids and Acylcarnitines kit #55000 for newborn screening (Chromsystems Instruments and Chemicals, Munich, Germany). A detailed description of the methodology is given in ref. [30]. Chromatographic separation of amino acids and acylcarnitines was performed on an Agilent 1210 LC system with a Prontosil 120-3-C18 AQ reversed-phase column (75 × 2.1 mm, 3 µm, Econova, Russia). The mass-spectrometric analysis was carried out in dynamic multiple-reaction monitoring (dynamic MRM) mode using an Agilent 6410 QQQ mass spectrometer (Agilent Technologies, Palo Alto, CA, USA). The mass-spectrometric analysis was carried out at the Core Facility of Mass Spectrometric Analysis at the Institute of Chemical Biology and Fundamental Medicine, the Siberian Branch of the Russian Academy of Sciences. Peak alignment, integration, and quantification were performed in the MassHunter Quantitative Analysis software (Agilent Technologies, Palo Alto, CA, USA). The concentrations of amino acids and acylcarnitines calculated from the ratio of peak area of an analyte to that of an internal standard were compiled into a table in the Excel file format for subsequent data analysis.

### 2.3. Statistical Analysis

Data processing, sparse partial least squares discriminant analysis (sPLS-DA), and visualization of the data were conducted in the R 3.6.1 scripting programming language in the RStudio 1.2.5001 environment using the mixOmics [35] and other packages. Differences in general characteristics between the studied groups were tested by the χ^2^ test and Mann–Whitney *U* test. Differences in amino acid and acylcarnitine levels between groups were evaluated by the Mann–Whitney *U* test. Data with *p* values less than 0.05 were regarded as statistically significant.

## 3. Results

### 3.1. Basic Population Characteristics

The basic characteristics of enrolled individuals are shown in Table 1. The prevalence of MetS in our study population of patients with schizophrenia was 38.4%, which is consistent with literature data [6,36,37]. Patients with MetS were older and had longer duration of schizophrenia and antipsychotic therapy; this result is explained by growing prevalence of MetS with age, which is typical for both the general population and patients with schizophrenia [38]. Furthermore, the additive effect of a long-term sedentary lifestyle and antipsychotic medication raises the risk of metabolic disturbances [39]. Our group of healthy volunteers matched our population of patients with schizophrenia in sex and age. According to Table 1, patients with schizophrenia comorbid with MetS are expected to have greater waist circumference and higher levels of glucose, triglycerides, high-density lipoprotein cholesterol, and blood pressure than patients with schizophrenia without MetS. Glucose levels in our patients with schizophrenia with MetS or without almost did not differ from reference values. This result is consistent with data from a large-scale meta-analysis showing that the main manifestations of MetS in schizophrenia are lipid metabolism disorders, not hyperglycemia [37].

### 3.2. Acylcarnitine and Amino Acid Levels in the Studied Groups

**Figure 1:**
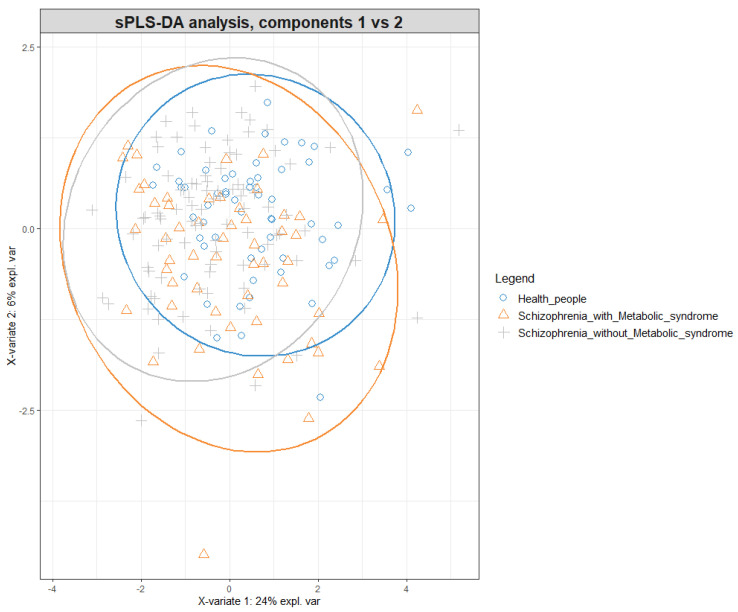
The scatter plot of 73 individuals on the basis of the first two principal components derived from concentrations of the assayed amino acids and acylcarnitines by sPLS-DA.

By sPLS-DA, we classified the tested serum samples into groups (Figure 1).

The levels of amino acids and acylcarnitines in patients with schizophrenia with or without MetS and in healthy individuals are presented in Table 2. Acylcarnitine C5, leucine/isoleucine, and alanine levels proved to be elevated in patients with MetS compared with the schizophrenia group without MetS. We detected lower concentrations of acylcarnitines C10 (*p* = 0.018 and 0.006, respectively), C10:1 (*p* = 0.009 and 0.003, respectively), C12 (*p* = 0.046 and 0.033, respectively), and C18 (*p* = 0.003 and 0.0001, respectively) in the patients with MetS and those without MetS in comparison with healthy controls. Additionally, in paranoid schizophrenia without MetS, there were lower levels of acylcarnitines C5 (*p* = 0.0001), C5:1 (*p* = 0.003), and C14 (*p* = 0.018) relative to healthy volunteers. The alanine concentration was statistically significantly higher in patients with paranoid schizophrenia comorbid with MetS as compared with healthy individuals (*p* = 0.044).

## 4. Discussion

Mostly, acylcarnitines’ levels did not depend on the presence of MetS; their medium- and long-chain species turned out to be underproduced as compared with the levels in healthy individuals. It is possible that these results can be attributed to a strong influence of long-term antipsychotic therapy. Kriisa et al. (2017) demonstrated upregulation of long-chain acylcarnitines and downregulation of short-chain acylcarnitines in drug-naïve patients with first-episode schizophrenia [31]. Nonetheless, in that research article, after 7 months of treatment with antipsychotics, there was a decrease in the level of long-chain acylcarnitines and an increase in concentrations of short-chain acylcarnitines accompanied by a weight gain in the patients [31]. These observations point to a change in energy metabolism under the influence of antipsychotics. Walss-Bass et al. [40] reported that in in vitro experiments, clozapine can oxidatively modify some enzymes of energy metabolism, in particular mitochondrial malate dehydrogenase, pyruvate kinase, and 3-oxoacid CoA transferase. Atypical antipsychotics suppress mRNA expression of carnitine palmitoyltransferase Ia [41]. Oh et al. [42] have demonstrated a decrease in the phosphorylation of AMP-activated protein kinase (a cellular protein kinase that controls the energy balance of the cell) and in the expression of several genes of fatty acid oxidation, e.g., carnitine palmitoyltransferase I. More recently, it was found that clozapine and olanzapine can cause underexpression of *Drp1* and *Mfn2* (responsible for changes in the shape of mitochondria), of genes of the electron transport chain, and of the corresponding proteins (i.e., enzymes) and hence a lower level of ATP in vitro [43]. Quetiapine reduces oxygen consumption and contributes to an uncoupling of the processes of respiration and phosphorylation [43].

We noted increased levels of an odd-chain acylcarnitine (C5), branched-chain amino acids (leucine/isoleucine), and their metabolite alanine in patients with schizophrenia comorbid with MetS as compared with those without MetS. Furthermore, we documented a trend toward higher valine levels in the patients with schizophrenia comorbid with MetS (*p* = 0.096) as compared with those without MetS. The C5 acylcarnitine is a derivative of leucine and isoleucine, whereas C3 is predominantly a product of valine and isoleucine breakdown after processing by branched-chain α-keto acid dehydrogenase [20,22]. An increase in levels of branched-chain amino acids is considered a predictor of insulin resistance [20,21], and in our patients, glucose levels were almost within the reference range. The observed trend toward higher valine levels in patients with schizophrenia comorbid with MetS (no statistical significance) may be associated with the beginning of the development of insulin resistance, which has not yet become widespread within the human body. By contrast, elevated levels of C5 and unchanged levels of C3 may be ascribed to impaired branched-chain α-keto acid dehydrogenase in patients with schizophrenia comorbid with MetS [22].

Our data are partly consistent with those of Cao B. et al. [30], who also documented the upregulation of odd-chain acylcarnitines in schizophrenia comorbid with MetS. At the same time, those authors reported an increase in the level of three medium-chain acylcarnitines and two long-chain ones in patients with schizophrenia comorbid with MetS, in contradiction to our study. It is likely that these discrepancies can be partly explained by differences between the study populations: for example, in Cao B. et al.’s study [30], ~30% of patients had first-episode schizophrenia, whereas in our case, there were no patients with first-episode schizophrenia and most patients had this disease for a long time. Therefore, the duration of treatment may also differ greatly between our studies. Given that schizophrenia is a heterogeneous disease, we included only patients with paranoid schizophrenia in the study, whereas the subtype was not specified in Cao et al.’s study [30]. This fact may also underlie the discrepancies between our studies.

In another study, Kriisa et al. [31] registered the growth of the ratio of C3 and C5 to free carnitine during an increase in the body mass index in patients with schizophrenia after antipsychotic therapy.

We believe that there is multidirectional participation of short-chain acylcarnitines in mental and metabolic disorders: underproduction of the former and overproduction of the latter (and vice versa for medium- and long-chain acylcarnitines). This notion is indirectly confirmed by the results of correlation analyses by other researchers revealing negative correlations between the level of acylcarnitines and levels of total cholesterol, triglycerides, and very low-density lipoprotein cholesterol as well as positive correlations with the level of high-density lipoprotein cholesterol in patients with mental disorders [30,44].

Because metabolic disorders during treatment with an antipsychotic begin to develop several weeks after the start of the therapy, this timing can “smooth out” alterations of the acylcarnitine profile. Taking into account the data of our study and the results of others, we propose that short-chain acylcarnitines may play a special role in the regulation of energy metabolism in patients with paranoid schizophrenia [30,31].

## 5. Conclusions

Thus, our data indicate deterioration of energy metabolism in paranoid schizophrenia, judging by a decrease in the concentration of most acylcarnitines. Aside from this alteration, in patients with paranoid schizophrenia comorbid with MetS, an increase in levels of valeryl carnitine (C5), leucine/isoleucine, and alanine was noticed, indicating their participation in the pathogenesis of metabolic disorders in paranoid schizophrenia.

## Figures and Tables

**Table 1:** Basic characteristics of the patients with schizophrenia and healthy individuals included in the analysis.

Parameter		Patients with MetS n = 39	Patients without MetS n = 73	Healthy Individuals n = 61
Gender	Female, n (%)	19 (48,8%)	38 (52.1%)	34 (55.7%) 27 (44.3%)
	Male, n (%)	20 (51.2%)	35 (47.9%)	
Age, years		40 (30; 48.5) ***	31 (26; 36.3)	35 (29; 46.2) **
Age of manifestation		26 (19.5; 30.5)	23 (20; 27)	Na
Duration of disorder, years		15 (8.5; 20) ***	7 (3; 12)	Na
PANSS, total score		92 (82.5; 107.5)	95 (86; 104)	Na
Duration of antipsychotic therapy, years		9 (4; 15) *	5 (2.5; 9.5)	
Total CPZeq		300 (175; 600)	324 (262.5; 600)	
Waist, cm		106 (96.5; 113.5) ***	83 (76; 90)	Na
Glucose, mmol/L		5 (4.65; 5.65) ***	4.7 (4.3; 5.1)	Na
Triglycerides, mmol/L		2.0 (1.77; 2.35) ***	1.1 (0.8; 1.4)	Na
HDL-C, mmol/L		0.82 (0.7; 1.0) ***	1.1 (0.9; 1.3)	Na
BP is above 130/85 mmHg (or with treatment of hypertension), n (%)		27 (69.2%) ***	14 (19.2%)	

Data are presented as a median (lower quartile; upper quartile); CPZ: chlorpromazine; HDL-C: high-density lipoprotein cholesterol; BP: blood pressure; na: not applicable; PANSS: Positive and Negative Syndrome Scale; * *p* < 0.05, ** *p* < 0.005, and *** *p* < 0.001 as compared with the schizophrenia group without MetS.

**Table 2:** Levels of amino acids and acylcarnitines in the patients with schizophrenia and healthy individuals included in the study.

Parameter, µM	Patients with MetS n = 73	Patients without MetS n = 39	Healthy Individuals n = 61	*p*-Value
C0	14.21 (12.95; 17.43)	14.29 (11.60; 16.61)	15.31 (12.54; 17.70)	*p*_0–1_ = 0.819 *p*_0–2_ = 0.080 *p*_1–2_ = 0.282
C2	0.687 (0.407; 1.307)	0.823 (0.679; 1.228)	0.764 (0.444; 1.136)	*p*_0–1_ = 0.276 *p*_0–2_ = 0.985 *p*_1–2_ = 0.241
C3	0.083 (0.068; 0.134)	0.088 (0.070; 0.115)	0.103 (0.062; 0.127)	*p*_0–1_ = 0.846 *p*_0–2_ = 0.176 *p*_1–2_ = 0.471
C3-DC	0.0216 (0.0137; 0.0292)	0.0231 (0.0137; 0.0304)	0.0245 (0.0170; 0.0349)	*p*_0–1_ = 0.804 *p*_0–2_ = 0.402 *p*_1–2_ = 0.689
C4	0.0543 (0.0464; 0.1046)	0.0632 (0.0479; 0.0856)	0.0758 (0.0579; 0.0933)	*p*_0–1_ = 0.412 *p*_0–2_ = 0.445 *p*_1–2_ = 0.974
C4-OH	0.0035 (0.0015; 0.0064)	0.0029 (0.0019; 0.0062)	0.0027 (0.0021; 0.0067)	*p*_0–1_ = 0.755 *p*_0–2_ = 0.957 *p*_1–2_ = 0.974
C4-DC	0.0093 (0.0062; 0.0106)	0.0080 (0.0055; 0.0093)	0.0089 (0.0076; 0.0096)	*p*_0–1_ = 0.214 *p*_0–2_ = 0.257 *p*_1–2_ = 0.141
C5	0.028 (0.021; 0.038)	0.022 (0.017; 0.029)	0.030 (0.022; 0.038)	*p*_0–1_ = 0.557 *p*_0–2_ = 0.0001 * p_1–2_ = 0.033 *
C5-OH	0.0044 (0.0034; 0.0078)	0.0057 (0.0044; 0.0075)	0.0060 (0.0048; 0.0077)	*p*_0–1_ = 0.145 *p*_0–2_ = 0.662 *p*_1–2_ = 0.263
C5:1	0.0027 (0.0020; 0.0045)	0.0025 (0.0018; 0.0036)	0.0045 (0.0037; 0.0065)	*p*_0–1_ = 0.126 *p*_0–2_ = 0.003 * p_1–2_ = 0.343
C5-DC	0.034 (0.020; 0.041)	0.029 (0.021; 0.035)	0.033 (0.027; 0.040)	*p*_0–1_ = 0.970 *p*_0–2_ = 0.779 *p*_1–2_ = 0.867
C6	0.0110 (0.0065; 0.0194)	0.0101 (0.0078; 0.0146)	0.0131 (0.0106; 0.0208)	*p*_0–1_ = 0.815 *p*_0–2_ = 0.066 *p*_1–2_ = 0.172
C8	0.0275 (0.0188; 0.0473)	0.0260 (0.0146; 0.0529)	0.0373 (0.0234; 0.0500)	*p*_0–1_ = 0.240 *p*_0–2_ = 0.138 *p*_1–2_ = 0.324
C8:1	0.0092 (0.0064; 0.0145)	0.0076 (0.0049; 0.0099)	0.0082 (0.0064; 0.0102)	*p*_0–1_ = 0.465 *p*_0–2_ = 0.146 *p*_1–2_ = 0.073
C10	0.027 (0.015; 0.055)	0.031 (0.014; 0.045)	0.045 (0.029; 0.066)	*p*_0–1_ = 0.018 * p_0–2_ = 0.006 * p_1–2_ = 0.990
C10:1	0.026 (0.014; 0.043)	0.028 (0.015; 0.037)	0.037 (0.027; 0.053)	*p*_0–1_ = 0.009 * p_0–2_ = 0.003 * p_1–2_ = 0.995
C12	0.014 (0.008; 0.017)	0.014 (0.007; 0.018)	0.016 (0.12; 0.24)	*p*_0–1_ = 0.046 * p_0–2_ = 0.033 * p_1–2_ = 0.886
C14-OH	0.0017 (0.0032; 0.0021)	0.0016 (0.0014; 0.0020)	0.0022 (0.0020; 0.0034)	*p*_0–1_ = 0.749 *p*_0–2_ = 0.075 *p*_1–2_ = 0.110
C14:1	0.0154 (0.0108; 0.0267)	0.0163 (0.0094; 0.0255)	0.0178 (0.0111; 0.0239)	*p*_0–1_ = 0.565 *p*_0–2_ = 0.544 *p*_1–2_ = 0.989
C14:2	0.0101 (0.0060; 0.0163)	0.0089 (0.0067; 0.0194)	0.0123 (0.0071; 0.0194)	*p*_0–1_ = 0.061 *p*_0–2_ = 0.127 *p*_1–2_ = 0.631
C16	0.0295 (0.0227; 0.0370)	0.0278 (0.0247; 0.0322)	0.0292 (0.0236; 0.0335)	*p*_0–1_ = 0.966 *p*_0–2_ = 0.146 *p*_1–2_ = 0.269
C16-OH	0.0014 (0.0012; 0.0022)	0.0013 (0.0010; 0.0016)	0.0015 (0.0011; 0.0019)	*p*_0–1_ = 0.708 *p*_0–2_ = 0.275 p_1–2_ = 0.273
C16:1	0.0107 (0.0061; 0.0151)	0.0097 (0.0081; 0.0132)	0.0116 (0.0066; 0.0161)	*p*_0–1_ = 0.954 *p*_0–2_ = 0.099 *p*_1–2_ = 0.136
C16:1-OH	0.0013 (0.0010; 0.0024)	0.0012 (0.0007; 0.0017)	0.0012 (0.0010; 0.0016)	*p*_0–1_ = 0.236 *p*_0–2_ = 0.942 *p*_1–2_ = 0.190
C18	0.0124 (0.0098; 0.0143)	0.0112 (0.0107; 0.0134)	0.0159 (0.0130; 0.0182)	*p*_0–1_ = 0.003 * p_0–2_ = 0.0001 * p_1–2_ = 0.864
C18-OH	0.0014 (0.0011; 0.0017)	0.0015 (0.0014; 0.0019)	0.0016 (0.0010; 0.0019)	*p*_0–1_ = 0.319 *p*_0–2_ = 0.733 *p*_1–2_ = 0.127
C18:1	0.057 (0.016; 0.065)	0.069 (0.045; 0.076)	0.059 (0.022; 0.084)	*p*_0–1_ = 0.649 *p*_0–2_ = 0.247 *p*_1–2_ = 0.724
C18:1-OH	0.0031 (0.0020; 0.0037)	0.0029 (0.0022; 0.0038)	0.0034 (0.0029; 0.0041)	*p*_0–1_ = 0.944 *p*_0–2_ = 0.985 *p*_1–2_ = 0.927
C18:2-OH	0.0025 (0.0011; 0.0029)	0.0013 (0.0008; 0.0022)	0.0017 (0.0011; 0.0023)	*p*_0–1_ = 0.207 *p*_0–2_ = 0.199 *p*_1–2_ = 0.061
Alanine	158.75 (131.80; 177.80)	138.84 (116.97; 168.80)	142.09 (114.92; 165.82)	*p*_0–1_ = 0.044 * p_0–2_ = 0.780 *p*_1–2_ = 0.019 *
Valine	88.36 (73.86; 96.57)	79.26 (69.24; 90.19)	83.4 (75.78; 95.18)	*p*_0–1_ = 0.456 *p*_0–2_ = 0.065 *p*_1–2_ = 0.096
Leucine/Isoleucine	49.58 (42.45; 58.58)	44.32 (32.81; 50.23)	44.72 (36.64; 51.67)	*p*_0–1_ = 0.098 *p*_0–2_ = 0.742 *p*_1–2_ = 0.019 *

Data are presented as median (lower quartile; upper quartile); *p*_0–1_: the comparison between the patients with MetS and healthy volunteers; *p*_0–2_: the comparison between the patients without MetS and healthy volunteers; *p*_1–2_: the comparison between the patients with and without MetS. * *p* < 0.05.

## Data Availability

Data are available on request, owing to privacy and ethical restrictions.
